# Semaphorins: Missing Signals in Age-dependent Alteration of Neuromuscular Junctions and Skeletal Muscle Regeneration

**DOI:** 10.14336/AD.2023.0801

**Published:** 2024-04-01

**Authors:** Damon Fard, Alessandra Barbiera, Gabriella Dobrowolny, Luca Tamagnone, Bianca Maria Scicchitano

**Affiliations:** ^1^Sezione di Istologia ed Embriologia, Dipartimento di Scienze della Vita e Sanità Pubblica,Università Cattolica del Sacro Cuore, 00168 Roma, Italy.; ^2^Fondazione Policlinico Universitario A. Gemelli IRCCS, 00168 Roma, Italy.; ^3^DAHFMO-Unità di Istologia ed Embriologia Medica, Sapienza Università di Roma, 00161 Roma, Italy.

**Keywords:** Aging, plexins, satellite cells, NMJs, semaphorins, regeneration, sarcopenia, YAP/TAZ

## Abstract

Skeletal muscle is characterized by a remarkable capacity to rearrange after physiological changes and efficiently regenerate. However, during aging, extensive injury, or pathological conditions, the complete regenerative program is severely affected, with a progressive loss of muscle mass and function, a condition known as sarcopenia. The compromised tissue repair program is attributable to the gradual depletion of stem cells and to altered regulatory signals. Defective muscle regeneration can severely affect re-innervation by motor axons, and neuromuscular junctions (NMJs) development, ultimately leading to skeletal muscle atrophy. Defects in NMJ formation and maintenance occur physiologically during aging and are responsible for the pathogenesis of several neuromuscular disorders. However, it is still largely unknown how neuromuscular connections are restored on regenerating fibers. It has been suggested that attractive and repelling signals used for axon guidance could be implicated in this process; in particular, guidance molecules called semaphorins play a key role. Semaphorins are a wide family of extracellular regulatory signals with a multifaceted role in cell-cell communication. Originally discovered as axon guidance factors, they have been implicated in cancer progression, embryonal organogenesis, skeletal muscle innervation, and other physiological and developmental functions in different tissues. In particular, in skeletal muscle, specific semaphorin molecules are involved in the restoration and remodeling of the nerve-muscle connections, thus emphasizing their plausible role to ensure the success of muscle regeneration. This review article aims to discuss the impact of aging on skeletal muscle regeneration and NMJs remodeling and will highlight the most recent insights about the role of semaphorins in this context.

## 1. Introduction

The skeletal muscle is the largest organ in the human body, representing 35-45% of the total mass [1]. It is characterized by a remarkable ability to adapt to physiological changes and to regenerate either in normal homeostasis or due to mechanical or pathological damage. This incredible plasticity is attributed to a small population of resident stem cells, representing 2% to 10% of all nuclei of a given fiber in healthy adult mammalian muscle. The latter are called satellite cells (SCs), due to their characteristic anatomical position on the surface of the muscle fibers, between the basal lamina and myofiber sarcolemma [2, 3]. SCs are quiescent and mitotically inactive in healthy conditions, but can rapidly reenter the cell cycle in response to specific growth signals or following damage. Once activated, the SCs proliferate and differentiate into myoblasts, which will subsequently merge with existing myotubes or form new myofibers in a few days [4, 5]. The regenerative process is characterized by a coordinated sequence of events, which resembles the process of embryonic skeletal muscle development, and it is dependent upon the serial expression of the myogenic regulatory factors (MRFs); the latter act in cooperation with specific transcription factors, such as Paired box protein 7 (Pax-7) and Myocyte Enhancer Factor 2 (MEF2), to maintain and preserve tissue structure and functionality upon injured stimuli [6, 7]. Nevertheless, in case of aging, extended injury, or pathological conditions, the complete regenerative program is severely affected, with a progressive loss of muscle mass and function, a condition known as sarcopenia [8-10]. It is likely that the compromised tissue repair program, upon aging or under pathological conditions, is dependent on either progressive loss of stem cell populations or missing molecular signals, which hinders the damaged tissues from efficiently carrying out the regenerative program. This may severely impact motor axon re-innervation and NMJs function and morphology, ultimately leading to skeletal muscle atrophy [11, 12]. Indeed, skeletal muscle regeneration is functionally successful only if the motor nerve terminal and a post-synaptic region of regenerating fibers are correctly connected by NMJ development, which is essential for functional contractility [13]. However, how neuromuscular connections are restored onto regenerating fibers is still largely unknown. In the last years, it was suggested that attractive and repelling signals used for axon guidance could be implicated in this process [14]; in particular, it has been demonstrated that certain semaphorins play a crucial role in this context [15, 16]. Semaphorins are a wide family of extracellular signaling molecules mediating cell-cell communication and a wide range of developmental and homeostatic processes [17, 18]. In skeletal muscle, a specific semaphorin signal, Sema3A, is involved in the restoration and remodeling of nerve-muscle connections, thus emphasizing its plausible role to ensure the success of muscle regeneration [19]. Several studies highlighted the role of another family member, Sema4C, in the regulation of the myogenin promoter driving myogenic differentiation [20]. Moreover, Sema6C is a poorly known transmembrane semaphorin unique in that skeletal muscle is the only peripheral tissue with high expression in the adult. Interestingly, Sema6C has been reported to be abundantly localized at the neuromuscular junction, while its expression is down-regulated following denervation [21].

This review aims to discuss the impact of aging and neuromuscular disorders on skeletal muscle regeneration and NMJs remodeling and highlight the most recent insights regarding the role of semaphorins in this context.

## 2. Skeletal muscle regeneration: an overview

Although skeletal muscle is considered a stable tissue with a little basal turnover of nuclei, it retains the ability to a rapid and extensive regeneration process in response to different injured stimuli [22, 23]. Muscle regeneration occurs in four interrelated and time-dependent phases: degeneration, inflammation, regeneration, and remodeling.

Degeneration - This is the first phase of the regenerative process, characterized by myofiber necrosis due to disruption of the sarcolemma that results in increased myofiber permeability. As a consequence, serum levels of muscle proteins normally retained in the myofiber cytosol, such as creatine kinase (CK) and troponin, are dramatically increased. Indeed, elevated serum levels of these molecules have been reported in humans and murine models muscle of degenerative diseases or mechanical trauma, making them important markers of muscle damage [24-26]. The acute necrosis with myofibers dismantlement is also related to an alteration of Ca^2+^ homeostasis, with the release of Ca^2+^ from the sarcoplasmic reticulum and an increased influx of extracellular Ca^2+^, ultimately leading to the activation of Ca^2+^- related proteolytic systems [27-30]. In particular, the Ca^2+^- activated cysteine proteases calpains initiate the proteolytic cleavage of muscle proteins driving tissue degeneration [31, 32].

Inflammation - The alteration of the muscular architecture, accompanied by the activation of the proteolytic pathways with the release of the intracellular content, favors the recruitment of tissue-resident mononucleated cell populations, mainly inflammatory cells, which provide chemotactic signals to circulating leucocytes that reach the site of damage through the bloodstream [33-35]. During the initial hours after muscle injury, the first mononucleated population which infiltrates the injured area is represented by neutrophils, then replaced by macrophages that become the main inflammatory cell type at the damaged site [36]. The macrophages infiltrating the injured tissue are represented by two distinct subpopulations sequentially involved in the process. Initially, the type M1 macrophages, secrete pro-inflammatory cytokines, including interleukin-1 (IL-1) and tumor necrosis factor (TNF), and play the essential role of cellular debris phagocytosis and digestion of necrotic fibers; subsequently, type M2 macrophages, secrete anti-inflammatory cytokines, such as IL-10, and thus promote the activation and proliferation of SCs [36-38]. In addition, the secretion of Fibroblast Growth Factor-6 (FGF-6), as well as the Insulin-like Growth Factor-1 (IGF-1), and the Hepatocyte Growth Factor (HGF), stimulates SCs activation and proliferation by initiating the proper regenerative phase [39-43].

Regeneration - Quiescent satellite cells are characterized by the expression of Paired box transcription factors (Pax3 and Pax7), and many other molecules including Neural Cell Adhesion Molecule (NCAM), M-cadherin (Mcad), Forkhead box protein K (FoxK), tyrosine-protein kinase Met (c-Met), Vascular Cell Adhesion protein 1 (VCAM-1), Sox 8, Sox 15, CD34, Integrins (α7 and β1), Syndecan 3 and 4, Caveolin-1, Calcitonin receptor (CTR), Lamin A/C, Emerin, and hairy/enhancer-of-split related with YRPW motif proteins and Heyl [44-56]. However, the progress of SCs from the quiescent phase to activation, commitment, and differentiation requires genetic and epigenetic adaptability to new biological tasks, resulting in dynamic alterations in the protein expression pattern. Furthermore, activated SCs induce the expression of proliferation and differentiation markers including desmin, Myogenic Factor 5 (Myf-5), and Myoblast Determination Protein (MyoD) while continuing to express Pax7, Mcad, VCAM1, caveolin 1, and integrin a7 [57-59]. Once quiescent SCs became activated, enter the cell cycle and start to proliferate, enabling the amplification of the myogenic cell pool [60, 61]. The transcription factor Pax7 is, in turn, able to induce the activation of the MRFs, including MyoD, Myf5, Myogenin, and MRF4, whose expression is finely and temporally regulated at various stages of the differentiation process [62, 63]. Specifically, in the proliferative stage, SCs are characterized by the expression of MyoD and Myf5 [34, 63]. In addition, the winged-helix transcription factor Myocyte Nuclear Factor (MNF), in particular the MNFα isoform, is transiently expressed selectively in proliferating SCs and it has been demonstrated its crucial role in muscle repair. Indeed, the lack of MNF severely affects SCs activation and proliferation and hampers the next steps of the regenerative process [64]. Many experiments have been focused on understanding the control of satellite cell function during muscle regeneration. One of the most important events is the release of nitric oxide at the injury site, which stimulates satellite cells’ proliferative activity [65]. Subsequently, damaged myofibers begin to secrete HGF that binds c-met receptors on satellite cells inducing their proliferation [66]. HGF is also secreted by satellite cells themselves through a positive feedback loop and induces the migration of satellite cells toward the site of muscle damage, thus amplifying the regenerative process [67]. As a result of proliferation, SCs differentiate into myoblasts that can either integrate with damaged fibers or fuse allowing the formation of new multinucleated myotubes characterized by centrally located nuclei and by the expression of the embryonic Myosin Heavy Chain (embryo-MyHC) [7, 34, 68]. The myoblast terminal differentiation is distinguished by the up-regulation of Myogenin and MRF4 and by the presence of nuclei arranged in the periphery of the newly formed muscular fibers [69, 70]. It has been suggested that in addition to SCs, other stem cells, and precursors, such as endothelial-associated cells [71], interstitial cells [72, 73], bone marrow-derived side population [74, 75], and fibroadipogenic progenitors (FAPs), can participate in muscle regeneration exerting a supportive role for SC activity [76]. These stem cell populations could either reside within the muscle or be recruited via circulation in response to homing signals emanating from the injured skeletal muscle. Among these progenitor cells, important support is given by bone-marrow-derived cells [77-80]. Indeed, transplantation of genetically marked bone marrow into immunodeficient mice revealed that marrow-derived cells migrate into areas of induced muscle degeneration, undergo myogenic differentiation, and participate in the regeneration of the damaged fibers [81].

Remodeling - Finally, the remodeling phase, which is a continuation of the repair process, is characterized by the reorganization of the muscle fibers (including the formation of forked fibers), their attachment to the surrounding ECM, and, not least, the formation of the NMJs with the restoration of muscle contractile function [82]. Indeed, successful regeneration and reshaping of NMJs are crucial to re-establish the functional capabilities and properties of skeletal muscle following damage [82].

## 3. Age-related alteration of skeletal muscle regeneration

The extraordinary regenerative capacity of skeletal muscle declines with aging [83-85]. Multiple factors may contribute to this phenomenon, including a decrease in the number and proliferative potential of satellite cells [86-88], telomere shortening within satellite cells [89], diminished innervation of senescent muscles [90, 91], increase in fibrotic tissue [92], and changes in the concentration of systemic and local growth factors and cytokines [93-97]. These events push aged SCs to a "point of no return," leading them into a pre-senescent state or triggering apoptosis [98]. Although several studies have reported opposite outcomes regarding the effect of aging in the number of satellite cells in mice [87, 99-103], it seems established that the decrease in the number of cells expressing satellite cell markers is the prevalent trend in humans [104-106]. Furthermore, the reduction in SCs number seems more pronounced in the aged fast-twitch extensor digitorum longus (EDL) muscle of rats compared to the slow-twitch soleus muscle, in agreement with the preferential loss and atrophy of fast-twitch fibers observed in sarcopenia [99]. However, despite an aging-related decreased number, the remaining resident SCs should be able to activate and sustain an adequate regenerative process. Indeed, it has been revealed that SCs retain their ability to respond to growth-promoting stimuli, undergo differentiation, fuse into myotubes, and produce a reservoir of cells throughout an individual's lifespan, suggesting that impaired regeneration may be due to aging-dependent alteration of the environment rather than inherent SC dysfunctions [99]. Heterochronic experiments have provided evidence that old muscles can effectively regenerate when transplanted into a young animal, whereas the regeneration of a young muscle transplanted into an elderly host is hindered [107, 108]. This hypothesis has been confirmed through parabiotic experiments, consisting in the connection of two organisms sharing a circulatory system, that demonstrated the rejuvenation of aged progenitor cells upon exposure to a youthful systemic environment [109]. These findings underscore the crucial role of the environment, which is influenced by circulating factors as well as the local secretome of factors released by various cells, including satellite cells and newly differentiating fibers. Additionally, the inflammatory context during the initial stages of muscle regeneration also contributes to this milieu [110]. Although numerous intracellular signaling pathways responsible for satellite cell activation and skeletal muscle fiber growth have been shown to undergo changes with aging, such as the FGF2 [111], TGF-β- [112], WNT pathways [113], JAK/STAT3 [10], p16INK4a [114, 115], and p38 [116, 117], recent studies have unveiled the involvement of key components of the Hippo signaling in this context. This pathway, known for its crucial role in tissue growth regulation in epithelial cells, has now been recognized for its contribution to adult skeletal muscle fiber growth and atrophy [118-121]. Indeed, it has been demonstrated that, as satellite cells proliferate, the activity of YAP and TAZ increases. Additionally, YAP/TAZ over-expression leads to an accelerated rate of myoblast proliferation; moreover, persistent YAP activation alone is sufficient to impair the terminal differentiation program necessary for proper myofiber fusion and maturation [122, 123]. Of note, YAP over-expression in in vivo experimental models results in the development of embryonic rhabdomyosarcoma-like tumors [123]; however, when YAP expression returns to basal levels, the observed phenotype is completely reversed. This indicates that temporary activation of the protein, either through physiological or pharmacological means, could potentially boost the proliferative capacity of satellite cells while still enabling a later differentiation of myoblasts to guarantee skeletal muscle growth and adaptation.

The skeletal muscle extracellular matrix has been shown to influence SC proliferation; in fact, when these cells are grown in vitro on a stiff medium their proliferative rate significantly decreases compared to an elastic medium [124, 125]. Notably, the extracellular matrix of skeletal muscles becomes more rigid with aging, due to extensive collagen crosslinking [126]. Since YAP and TAZ function as mechanotransducers [127], the aging-dependent increase of the extracellular matrix rigidity may impact their activity in fibroblasts and SCs. Indeed, it has been demonstrated by Stearns-Reider et al. that a stiff extracellular matrix enhances YAP translocation in the nucleus of fibroblasts [128]. Consequently, YAP activity in fibroblasts promotes the fibrogenic conversion of skeletal muscle, creating a feedback loop whereby fibrogenic conversion leads to further stiffening of the extracellular matrix. In addition, SCs have been shown to regulate the production of extracellular matrix by fibroblasts [129] and, despite a decline in SC abundance in aged skeletal muscles, their presence might still be involved in increasing extracellular matrix production and rigidity [130]. It remains uncertain whether the stiffness of the matrix also affects YAP levels in SCs. Nevertheless, aged muscle stem cells experience a decrease in the formation of focal adhesions, which alters the cytoskeletal properties of the muscle alongside increased YAP localization in the nucleus [130]. Although YAP expression facilitates the proliferation of SCs, it also promotes fibrogenesis in fibroblasts, thus the overall benefit of increasing YAP expression may depend on the conditions.

All in all, based on current literature, it is hard to speculate whether a modulation of TAZ/YAP signaling could represent a potentially therapeutic approach to improve aged-dependent alterations of skeletal muscle regeneration [131]. Thus, this important issue awaits experimental clarification in preclinical models.

## 4. Age-related alterations of NMJ morphology and function

The age-related alteration of skeletal muscle regeneration may have important repercussions on motor axon re-innervation and NMJs function and morphology, ultimately leading to skeletal muscle atrophy [11, 12, 132]. Indeed, in healthy conditions, the typical “pretzel-shape” endplate is the result of the motor neuron terminal end arborization. This structure is enlarged and forms pre-synaptic boutons, which contain synaptic vesicles filled with the neurotransmitter acetylcholine (ACh). Boutons directly overlie post-synaptic invaginations of the sarcolemma called junctional folds [133], upon which high-density clusters of acetylcholine receptors (AChRs) reside. Once released into the synaptic cleft, ACh binds to AChRs, determining a local depolarization that then propagates throughout the muscle fiber. Voltage-dependent calcium channels open once an action potential hits the pre-synaptic component, therefore, permitting calcium to initiate the delivery of ACh in the synaptic cleft. AChR in the post-synaptic membrane is activated by ACh and results in the development of an action potential, triggering the stimulation of voltage-gated dihydro-pyridine receptors (DHPRs) in the sarcolemma and ryanodine receptors (RyRs) in the membrane of the sarcoplasmic reticulum. It has been reported that in mammal muscles there are three types of neuromuscular synapses, slow, fast fatigue-resistant, and fast-fatigable. Each type is associated with its own muscle fiber subtype that can be recognized by the expression of specific myosin heavy chain (MHC) protein isoforms, type I, type IIa or type IIb, and IIx, respectively. These motor units differ in their physiological properties, anatomical plasticity, and susceptibility to loss of neuromuscular connectivity [134-136]. Specialized glial cells identified as terminal Schwann cells (tSCs) coated the nerve terminal and generate a basal lamina that merges with a muscle fiber basal lamina at the boundary of the NMJ [133, 137-139]. TSCs play an important role in the plasticity, maintenance, and regeneration of NMJs. Indeed, following muscular denervation tSCs processes serve as a guidance substrate for regenerating motor nerve sprouts and can elicit new nerve sprouting at neighboring NMJs following partial denervation [140, 141]. Furthermore, fibroblast-like cells called kranocytes develop a loose envelope on the NMJ and take part in nerve repair and regeneration, making them additional important factors in the operation of the neuromuscular network [142, 143].

During aging, NMJs undergo dramatic morphological, functional, and molecular changes in both pre-synaptic and post-synaptic regions that lead them to ultimately degenerate [144-146].

Notably, at the level of the pre-synaptic region, increased axon diameter and larger nerve terminal area have been observed [147, 148]. However, this effect is not accompanied by increased ACh stores, since it has been demonstrated that as presynaptic branching increases with age, the quantity of available ACh declines [149, 150]. At the post-synaptic level, the endplates decrease in size and are fragmented with a gradual decrease in the number of AChRs per junction, and the number and length of postsynaptic folds are reduced, leading to a functional impairment of NMJ response [132, 144-147, 149-152]. Additionally, tSCs relocate from the motor nerve terminal and protrude branches toward the synaptic cleft, which leads to the neuromuscular system functional drop by aging [153, 154]. The diminished ability of aged motor axons to effectively reestablish connections with muscle fibers after periods of degeneration [155] contributes to the muscle fibers atrophy and the loss of motor neurons units, leading to an age-related accumulation of intermuscular fat and fibrous tissue [156, 157], a minor degree of muscle fiber clustering [153], and a decrease in muscle size [158, 159]. Several trophic factors including neurotrophins, cytokines, and growth factors, have been implicated in the development of pre- and post-synaptic structures, as well as in preserving neuronal and synaptic plasticity at the NMJ [160, 161]. Although the precise involvement of these factors in promoting NMJ maintenance in the context of aging has not been fully determined, recent studies suggest that a variety of trophic factors, including brain-derived neurotrophic factor (BDNF), neurotrophin-3 (NT-3), neurotrophin-4 (NT-4), cytokines such as glial-derived neurotrophic factor (GDNF) and ciliary neurotrophic factor (CNTF), as well as other growth factors such as insulin-like growth factor (IGF-1 and IGF-II) and fibroblast growth factors (FGF), all exert modulatory effects on the neuromuscular system to a different extent during the aging process [152, 162].

## 5. Semaphorin family of guidance cues

**Figure 1.**
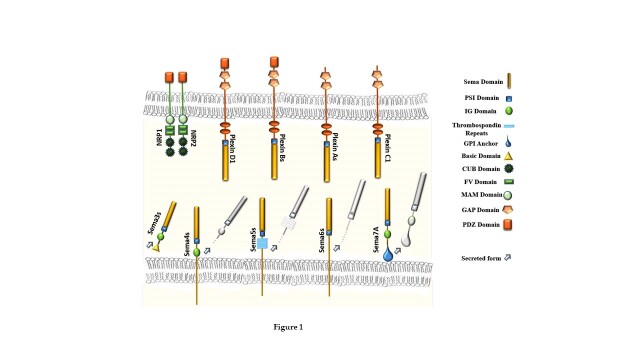
Schematic representation of the structural arrangement of Semaphorin subclasses expressed in vertebrates and their associated receptor subsets.

Semaphorins constitute a large family of extracellular signaling molecules, conserved across animal species, with a multifaceted role in cell-cell communication. Originally discovered as axon guidance factors, recently semaphorins have been implicated in the regulation of immune responses, cancer progression and angiogenesis, organ formation, innervation of skeletal muscle, and a variety of other physiological and developmental functions [17, 163]. Over twenty semaphorin proteins have been identified so far. They can be divided into eight classes (Sema1-7, and the viral-encoded members SemaVs), based on phylogenetic relationships and structural features [164-166]. Sema1, Sema2, and Sema5C are found in invertebrates. Among family members expressed in vertebrates, class 3 semaphorins are secreted, while others are transmembrane or membrane-bound through a GPI anchor, although they can be cleaved and shed in the environment in soluble form [17, 163, 167] (Fig. 1). Semaphorins are found in practically all tissues and their expression varies dramatically with age. During development, neuronal and non-neuronal cells express a wide range of semaphorins. In addition, semaphorins are found in the circulatory, endocrine, gastrointestinal, hepatic, immunological, musculoskeletal, renal, reproductive, and respiratory systems [16, 164].

The Plexin family comprises the main receptors for semaphorins; they are large single-pass transmembrane molecules that contain an extracellular semaphorin-binding domain. [17, 167]. There are nine plexin family members that can be divided into four groups, plexins A-D. While transmembrane semaphorins can directly activate the plexins, most secreted class-3 semaphorins rely on neuropilins as co-receptors [168] (Fig. 1).

Transmembrane Semaphorins are capable of bi-directional signaling and can interact both in-trans- and in-cis with plexin counterparts, resulting in distinct and tightly controlled signaling cascades. It has been shown that the conventional semaphorin/plexin “forward” signaling can affect cytoskeletal reorganization, cell adhesion, and cell migration. Semaphorin may also trigger so-called “reverse” signaling cascades through their cytoplasmic tails, acting as receptors rather than ligands [169, 170].

Small GTPases including R-Ras, Rap1, and RhoA, which modulate cell-substrate adhesion and cytoskeletal dynamics, are the major effector molecules in Sema/Plexin signaling pathways. Furthermore, sema-plexin signaling has been found to control receptor-type and cytosolic tyrosine kinases (e.g. Met, ErbB2, Abl), as well as downstream effector pathways like PI3K/AKT and MAPK/ERK [164]. Semaphorins have recently been shown to regulate gene expression reprogramming, for example, Sema4C can activate the SMAD1/5 and ID1/3 transcriptional regulators [171], and Sema6C recruits non-receptor tyrosine kinase c-Abl and regulates focal adhesion kinase (FAK), promoting nuclear localization of YAP [172].

## 6. Semaphorin signals in Skeletal Muscle

Skeletal muscle is one of the human body's most active and flexible tissues. Muscle mass loss, mostly determined by an unbalance between protein synthesis and breakdown, is influenced by several factors such as increased levels of oxidative stress, chronic inflammation, and alteration of hormone levels, which often occur during aging and in several pathological disorders [173-175]. In these conditions, not only the regenerative process is compromised but also NMJ degeneration may occur, seemingly linked to the failure of muscle re-innervation [132]. As mentioned above, the NMJ is the connection between a motor neuron's nerve terminal and its target muscle fiber, responsible for converting electric signals propagated along motor axons into muscle contraction [176]. Stabilizing motor-muscle connections is essential for maintaining muscle tone and triggering movement [15, 177]. A denervating injury causes a regenerative response, in which both intrinsic and extrinsic stimuli act on motor nerve terminals to promote growth and axonal sprouting, resulting in muscle fiber reinnervation [178].

Axon guidance molecules, including Semaphorins, have been widely suggested to play a role in peripheral nerve guidance and regeneration following damage, owing to their involvement in controlling target innervation during development [15, 17]. In fact, diverse semaphorins, and their receptors in the plexin and neuropilin families, have been found to play distinctive roles in axonal navigation and in structuring the neuromuscular system. For example, in the fly *D.melanogaster*, Sema1 acts as a dose-dependent repelling signal for motor axons [179] and Sema2 as a muscle-derived inhibitor of synapse formation [180, 181]. In mammals, the secreted semaphorin 3A (Sema3A) controls axonal guidance and growth [182] as well as cell migration [183], while Sema4D is crucial in the development of skeletal muscle and hindbrain boundary architecture [184], and Sema6C plays a crucial role in neuromuscular communication [21].

**Figure 2.**
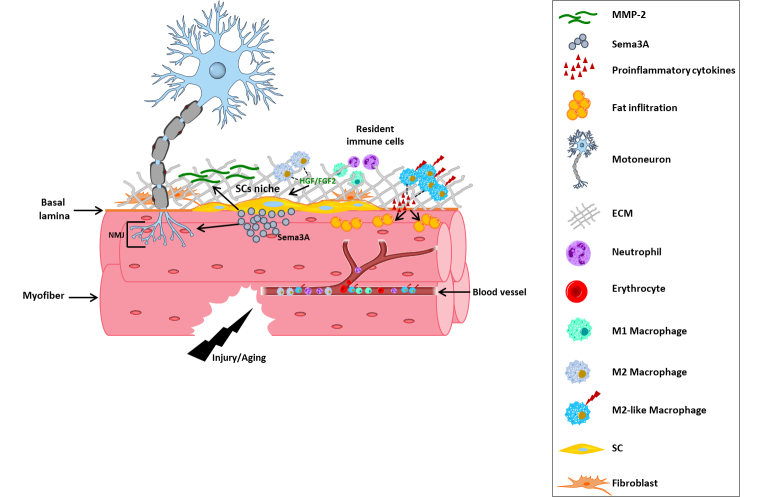
Schematic representation of Sema3A activity on skeletal muscle regeneration and NMJ maintenance.

## 7. Age-dependent alteration of Semaphorins in skeletal muscle regeneration and NMJ remodeling.

Sema 3A. Several studies aimed at elucidating the role of semaphorins expressed in skeletal muscle in the regeneration process. In 2009, Tatsumi et al. demonstrated that SCs, which are particularly localized at the NMJs (20-fold greater abundancy than in non-junctional regions) secrete Sema3A in response to muscle damage. Interestingly, the timing of Sema3A expression is related to the different phases of the regenerative process. In particular, in the early phases of the regenerative process, after a crush injury of the lower hind-limb muscle in the adult rat, it was observed the upregulation of Sema3A mRNA and Sema3A levels, in concomitance with increased levels of myogenin; subsequently, Sema3A expression returned to the basal levels in the final steps of the differentiative process [185]. The same expression profile of Sema3A was found in myogenic precursor cells in culture [186, 187]. Sema3A secretion by SCs is induced by several growth factors released in the tissue interstitium, including HGF and FGF2, which are implicated in SC activation after muscle damage [185]. In the effort to clarify the paracrine source of HGF, Sakaguchi et al. demonstrated that activated anti-inflammatory macrophages (M2), playing a crucial role in the regenerative process, produce HGF and thereby promote SCs chemoattraction and Sema3A expression. These data support the idea that M2 controls the spatial and temporal increase of Sema3A levels by releasing HGF, which subsequently triggers a surge in Sema3A secretion by SCs recruited at the injury site. This mechanistic model might guarantee a delay in the reconnection of motoneuron terminals to damaged fibers during the initial stages of muscle regeneration, thereby coordinating the restoration of muscle fiber integrity with the resolution of inflammation induced by injury [188]. These data extend the well-characterized role of SCs in muscle regeneration and repair to a new crucial role in controlling myofiber re-innervation [189] (Fig. 2). In a recent paper by van Beek et al. it was demonstrated that a population of atypical M2-like macrophages producing proinflammatory cytokines appeared to accumulate in skeletal muscle during aging, resulting in muscle fat infiltration and promotion of cell senescence [190]. Although further studies are required to fully elucidate the role of M2-like macrophages during injury repair in aged skeletal muscle, it is known that the alteration of macrophage polarization impairs the repair program, eliciting a fibrotic response [50,125].

In addition, as mentioned above, aging affects SC reservoir and its crosstalk with the environment, thus impairing the regeneration process in the elderly. Since SCs and M2-like macrophages are involved in the regulatory circuit of NMJs regeneration it is possible to speculate that aged-associated alteration of their function might compromise Sema3A release by SCs, and the consequent spatial and temporal restoration of muscle-nerve communication.

It was underlined above the importance of NMJs alteration during aging, these fundamental structure are commonly studied in experimental models of muscle denervation which have revealed that Terminal Schwann cells (covering motor nerve endings) are critical in the maintenance and regeneration of NMJs [154]. Interestingly, in 2006, De Winter et al. showed in a rat model, upon gastrocnemius muscle denervation by sciatic nerve injury, that Sema3A is selectively upregulated in terminal Schwann cells at NMJs of subtype IIb/x muscle fibers, but not type I and IIa fibers; these findings indicated that the microenvironment controlling the re-innervation process is differentiated at the level of single muscle fiber [191]. Indeed, it is known that slow-twitch oxidative fibers are more resistant to damage, and to a variety of atrophic conditions, compared to fast-twitch glycolytic fibers [192]. The findings above suggested that the expression of Sema3A by terminal Schwann cells not only suppresses nerve terminal plasticity at specific neuromuscular synapses, but may also contribute to their early and selective loss in physio-pathological conditions, including aged-related sarcopenia, cachexia, muscular dystrophies, and ALS, where the fastest muscle phenotype is more severely compromised if compared with slow-twitch muscles [193, 194].

Apparently in contrast with the data reported by De Winter, it was later observed by Shadrach et al., in a mouse model of peroneal nerve injury, that Sema3A is basally expressed in uninjured fast-twitch EDL muscle, while a noteworthy reduction of its levels was noted following denervation [15]. Several aspects should be investigated further to elucidate these apparent discrepancies. While the first study demonstrated Sema3A highly localized distribution in Schwann cells by in situ analysis, the latter quantified gene expression in muscle lysates, which may reflect diffuse low Sema3A expression in muscle (or other) cells, possibly curbed by denervation. Additional distinctive issues could be related to species-specific variations between the two rodent models, or signaling pathways differentially involved in the innervation of muscles of the posterior (gastrocnemius) and anterior (EDL) leg compartments, due to dorso-ventral embryonic patterning. It is worth mentioning that data from both studies similarly imply that Sema3A signaling plays a more prominent role in fast-twitch muscle fibers. In fact, although Sema3A alterations in injured soleus muscle were not investigated, significantly lower mRNA levels at baseline were detected in this muscle compared to the fast-twitch EDL [15]. The reasons behind discrepancies in Sema3A expression among different muscle groups, and their potential significance, remain an unresolved matter.

Sema3A-dependent fiber-type regulation was shown to impact on fiber maintenance, survival, and remodeling in mature muscle. In particular, Suzuki et al. demonstrated that, in myotube cultures derived from mouse satellite cells, Sema3A knockdown leads to reduced expression of myogenin and myosin heavy chain type I (the isoform specifically expressed by slow-twitch, fatigue resistant-fibers) and increased levels of myosin heavy chain type II (specifically expressed by the fast-twitch fibers) [187]. These findings underline another important activity mediated by Sema3A, in addition to neuro-muscular remodeling, i.e., the regulation of fiber-type distribution in skeletal muscle; this may furthermore suggest a crucial role of Sema3A in the maintenance of the slow fiber phenotype, favoring a more resistant muscular environment, which is known to be severely compromised in aging and in pathological conditions.

In addition to SCs that express Pax7, a population of interstitial muscle progenitors characterized by the expression of the bHLH transcription factor Twist2 (Tw2) was recently found in skeletal muscle [195]. These cells are anatomically and transcriptionally different from Pax7^+^ SCs that are localized beneath the basal lamina in all myofiber types. Indeed, Tw2^+^-cells fuse only with type IIb/x fast-twitch myofibers and genetic ablation of Tw2^+^-cells in mice results in selective atrophy of type IIb myofibers [195]. Moreover, in 2017, Liu et al. demonstrated that ectopic expression of Twist2 totally represses the myogenic program and activates genes involved in cellular migration and matrix degradation, highlighting the role of Tw2 in regulating both myogenesis and tissue invasiveness [195]. The invasiveness of Tw2^+^-cells is also highlighted by recent discoveries that identified an increased expression of Tw2 gene in rhabdomyosarcoma, a highly invasive tumor type of muscle origin [196].

**Figure 3.**
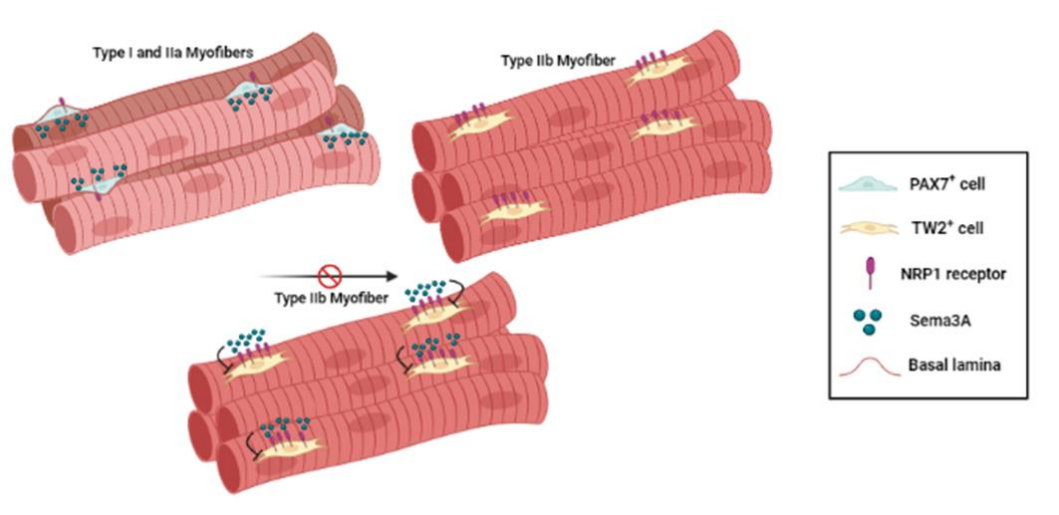
Schematic representation of the selective avoidance of Sema3A by Tw2-derived myoblasts but not by Pax7-derived myoblasts, as result of the differential expression of NRP1.

The specificity of Tw2^+^ progenitors in the formation of type IIb myofibers has been recently linked to Sema3A expression. Indeed, Tw2^+^-cells exhibit NRP1 receptor for Sema3A at higher levels compared to Pax7^+^-cells, thus identifying NRP1 as a marker of Tw2^+^-cells [197]. In addition, Sema3A released by type I and type IIa myofibers acts as chemorepellent for Tw2^+^-cells, fostering their fusion with type IIb myofibers (lacking Sema3A). Indeed, over-expression of Sema3A in type IIb myofibers is sufficient to prevent Tw2^+^-cells and type IIb myofibers fusion in vivo. On the other hand, Pax7^+^-SCs cells, carrying low levels of NRP1, are not repelled by type I or IIa muscle fibers, showing no fiber-type specificity. These evidences underline the presence of an intercellular signaling mechanism by which Twist2 controls the expression of NRP1 in Tw2^+^-cells, conferring myofiber specificity to Tw2^+^-cells fusion, gated by the presence of the repelling cue Sema3A (Fig. 3). To our knowledge, Tw2^+^ progenitors represent the first example of a fiber-type specific myogenic progenitor population. Type IIb fibers are the most prevalent and are particularly vulnerable to injury and diseases in mice. Thus, it becomes crucial to preserve the size and integrity of type IIb fibers during the aging process, and in this context, the manipulation of the Sema3A-NRP1 signaling could represent a valuable approach to preserve the fastest muscle phenotype in elderly or neuromuscular pathologies.

It is worth noting that semaphorins are not only implicated in axon guidance and growth but are also in many other processes directly connected to successful muscle regeneration such as inflammation, angiogenesis, and cell migration [198]. As detailed in Section 2 of this review, injury of myofibers results in rapid necrosis with consequent activation of an inflammatory response [60] and it has been reported that Sema3A can alter vascular permeability as well as T-cell migration [199, 200]. Indeed, a series of experiments in culture showed that Sema3A acts to reduce endothelial cell survival by inhibiting VEGF and integrin activities, required for vessel formation [201-203]. Thus, it is possible to speculate that in the site of damage, Sema3A could prevent the onset of an immune response modulating the regenerative process [189].

In addition, this semaphorin is involved in the activation of matrix metalloproteinase (MMP)-2 activity in skeletal muscle. The family of MMPs, including MMP-2 whose secretion resulted increased during myogenesis, is involved in ECM morphogenesis, the release of signaling molecules from the ECM, shedding of membrane-associated proteins, and cell motility, all indispensable processes for the remodeling phase of skeletal muscle regeneration [204-207]. Sema3A regulation was furthermore associated with Amyotrophic Lateral Sclerosis (ALS). Indeed, it has been detected a consistent upregulation of Sema3A levels in the motor cortex of ALS patients, and in situ hybridization localized Sema3A expression in motor neurons. Moreover, the rise of Sema3A has been associated with axonal loss and tissue regeneration failure in ALS patients [208].

Sema 4D. Transmembrane Semaphorins have been reported to play a role in skeletal muscle embryonic growth. For instance, Sema4D is crucial in the development of skeletal muscle and hindbrain boundary architecture, and its silencing caused abnormalities in the hindbrain and trunk anatomy of zebrafish embryos at all developmental stages. In embryos subjected to Sema4D knock-down it was also observed a large increase in cellular apoptosis but no noticeable decrease in proliferation. Furthermore, in Sema4D-morphants it was found an abnormal expression of three hindbrain rhombomere boundary elements: wnt1, epha4a, and foxb1.2, as well as of two myogenic regulatory factors, MyoD and myogenin; this implies Sema4D in a complex gene regulation network in hindbrain development, which includes Eph-ephrin, Wnt, and possibly Notch pathways [184]. Future experiments are required to validate the importance of Sema4D in adult mammalian models.

Sema 4C. Another related molecule, Sema4C, regulates myogenin promoter activity in the course of myogenic differentiation. In fact, during the differentiation of C2C12 mouse myoblasts, as well as upon injury-induced skeletal muscle regeneration, Sema4C expression increases significantly. Stable or transient expression of Sema4C in C2C12 leads to enhanced myogenic differentiation, as revealed by hastened myotube production and the expression of muscle-specific proteins. Notably, Sema4C overexpression leads to p38 phosphorylation, and the application of a p38 inhibitor or dominant-negative can block Sema4C-driven functional effects. Conversely, Sema4C silencing during C2C12 myoblast differentiation suppressed p38 phosphorylation and resulted in significantly reduced myotube formation [20]. It remains to be demonstrated if the treatment with soluble forms of Sema 4C could be used to promote regeneration in aged muscle.

Sema 6C. Sema6C is another transmembrane semaphorin found to have a role in neuromuscular communication. In particular, Sema6C mRNA and protein levels were found to be down-regulated in denervated mouse hindlimb and hemidiaphragm muscles, compared to controls. It has been shown that, indeed, Sema6C immunoreactivity is concentrated near NMJs suggesting its role in neuromuscular transmission [21]. Very recently it has been demonstrated by Fard et al. that Sema6C exerts a growth-promoting activity in cancer cells through the upregulation of FAK, ERK and YAP/TAZ signaling [172]. Notably, as previously mentioned YAP/TAZ have been implicated in skeletal muscle proliferation and differentiation [118, 122, 209-211], and YAP mutations inhibits NMJ regeneration after nerve injury, indicating a role of muscle YAP in this process [212]. Yet, it has been reported that in young subjects, YAP protein level is higher in slow-twitch muscle fibers than in fast-twitch fibers and its expression is ~50% lower in both these muscle fiber types in aged subjects compared to younger controls. These data suggest possible fiber type-dependent differences in the regulation of YAP, and that a reduction in YAP could play a role in the age-dependent loss of skeletal muscle mass [213]. These observations allow speculation that Sema6C-dependent YAP/TAZ pathway activation might be involved in the regenerative process and in the maintenance of NMJ, in both physiological and pathological conditions.

## 8. Therapeutic implications and future directions

Defective tissue repair upon aging or under pathological conditions is due to either progressive loss of stem cell populations or altered extracellular signals that hinder damaged tissues from efficiently carrying out the regeneration program. Indeed, skeletal muscle regeneration is functionally successful only if the motor nerve terminal and a post-synaptic region of regenerating fibers are correctly connected by establishing NMJ, which is essential to resume functional contractility. A deeper understanding of the mechanisms underlying dysfunctional muscle regeneration and NMJ remodeling is warranted, in order to identify novel therapeutic targets for counteracting the negative impact of aging on the neuromuscular system. Several studies have shown that the skeletal muscle has a significant secretory function, recently elucidated by proteomic studies. Notably, some of these secreted proteins, known as myokines, act locally on muscle cells through autocrine/paracrine loops, and on nearby tissues like muscle arteries; moreover, they may be released into the bloodstream deploying effects on the entire body. Among these factors, specific semaphorins have been unexpectedly implicated in the restoration and remodeling of the nerve-muscle connections, thus emphasizing their plausible role to ensure the success of muscle regeneration. For example, Sema3A has been identified as a key player in the regenerative process, with its expression being tightly regulated during different phases of muscle repair. Sema3A is secreted by satellite cells (SCs), which are highly concentrated at NMJs and respond to muscle damage by upregulating its expression. Targeting Sema3A activity, either directly or via its receptors, could potentially enhance the coordinated onset of fiber integrity renewal and inflammation resolution during muscle regeneration. Additionally, terminal Schwann cells at NMJs specifically express Sema3A, suggesting its involvement in the maintenance and regeneration of NMJs. Indeed, understanding the signals governing the complex interplay between SCs, macrophages, and Schwann cells in muscle regeneration may lead to future therapeutic strategies for aging-related muscle decline, neuromuscular pathologies, and muscle phenotypic regulation. Other studies have furthermore unveiled the potential importance of transmembrane semaphorins Sema4D, Sema4C, and Sema6C in muscle development, differentiation, and neuromuscular communication. Moreover, semaphorins are known to regulate inflammatory cell recruitment and activity and new vessel formation, which are crucially involved in successful muscle regeneration. Harnessing the potential of semaphorins in modulating these processes could pave the way to the validation of novel targets with therapeutic potential for the treatment of aging and neuromuscular disorders characterized by muscle atrophy and impaired regeneration.
